# Human Activities Have Reduced the Potential Distribution of Cotton in Xinjiang, but Climate Change Is Expected to Expand Its Future Suitable Area

**DOI:** 10.3390/plants15111622

**Published:** 2026-05-25

**Authors:** Jie Li, Shanwei Lou, Pengzhong Zhang, Tengfei Ma, Paerhati Maimaiti

**Affiliations:** 1 Cotton Research Institute of Xinjiang Uyghur Autonomous Region Academy of Agricultural Sciences/National Cotton Engineering Technology Research Center, Urumqi 830091, China; lj880902@xaas.ac.cn (J.L.); wei.lou@163.com (S.L.); zhangpz@163.com (P.Z.); 2 Xinjiang Key Laboratory of Cotton Genetic Improvement and Intelligent Production/Xinjiang Cotton Technology Innovation Center, Urumqi 830091, China

**Keywords:** Xinjiang cotton, climate change, human activities, ensemble model, niche analysis

## Abstract

Cotton is a vital cash crop that underpins regional agricultural systems and the global textile supply chain. However, climate change and increasing human activity are reshaping the spatial distribution of areas suitable for cotton cultivation, with the potential impacts being particularly pronounced in arid and semi-arid regions. This study integrated high-resolution cotton distribution data, environmental variables and human activities and employed ensemble model and niche analysis methods to systematically assess cotton suitability in Xinjiang under current and future climate scenarios. The results indicate that the ensemble models demonstrate high predictive performance, with both model types (Model 1: Environmental; Model 2: Environmental and human activity) achieving AUC values exceeding 0.97 and TSS values exceeding 0.84. Under current climatic conditions, suitable cotton-growing areas are primarily distributed on both sides of the Tianshan Mountains, and the inclusion of human activity factors results in a 13.71% reduction in suitable area. Moreover, Future climate change is projected to result in an increase in its suitable range of between 28.25% and 94.10%, with the most significant expansion occurring under the high-emissions scenario. MESS analysis indicates that the newly identified suitable areas in the future bear a high degree of similarity to current environmental conditions, whilst MOD analysis further highlights that temperature and precipitation are the key drivers of environmental variation. Additionally, Xinjiang cotton will retain a high degree of ecological niche under future climatic conditions. These findings provide important scientific evidence for optimizing the spatial distribution of cotton cultivation in Xinjiang and for climate-adaptive agricultural management.

## 1. Introduction

Global climate change and the intensification of human activities are posing unprecedented challenges to agricultural ecosystems, with significant changes occurring in crop growing conditions and spatial distribution patterns [1,2,3]. Particularly in arid and semi-arid regions, the distribution of water resources, temperature gradients and land-use patterns have a profound impact on crop suitability [4,5]. Cotton is one of the most important economic crops globally, and its fiber yield and quality not only influence the development of the textile industry but also serve as a critical foundation for agricultural economies and regional grain–cotton security [6]. Xinjiang is China’s largest cotton-producing region, with both its yield per unit area and total output ranking first in the country [7]. According to data from the National Bureau of Statistics of China, in 2025, Xinjiang’s cotton-sown area was 38.875 million mu, with a total output of 6.165 million tonnes, accounting for 86.99% and 92.83% of the national total respectively [4,8]. Therefore, a scientific assessment of the potential suitability of Xinjiang cotton under current and future climate change scenarios is of significant theoretical and practical importance for the rational planning of cropping patterns, the optimization of irrigation management, and the safeguarding of regional agricultural production security.

Xinjiang is characterized by a typical continental climate, with a vast territory and complex topography, where temperature and precipitation exhibit pronounced spatial heterogeneity [9]. The distribution of cotton cultivation is influenced by a combination of factors, including altitude, slope, soil type, water resources and human management activities, resulting in a spatially uneven distribution of cultivation [10,11]. Previous studies on the distribution of cotton in Xinjiang have primarily relied on statistical annual reports, field surveys and remote sensing imagery [7,9]. Whilst these methods can reflect historical cropping patterns, they lack a comprehensive analysis of high-resolution environmental data, making it difficult to accurately predict the potential impact of future climate change on the spatial distribution of cotton [5,10]. Furthermore, traditional research has typically focused on changes in yield and cultivated area, whilst systematic studies on niche dynamics, quantitative predictions of areas of potential suitability, and the role of human activities in distribution patterns are lacking [12,13]. At present, studies applying species distribution models to cotton in Xinjiang remain relatively limited, particularly those that conduct systematic analyses of dynamic suitability projections and niche shifts under future climate scenarios. This research gap limits our in-depth understanding of the mechanisms underlying the evolution of cotton distribution patterns and their response to climate change, and to some extent also affects the practical value of the relevant research findings in agricultural planning and management. Therefore, it is necessary to integrate high-resolution spatial data with multi-source environmental variables to conduct systematic analyses under future climate scenarios, thereby enabling more accurate assessments of the potential suitability and spatial distribution dynamics of cotton in Xinjiang.

In recent years, species distribution models (SDMs) have become important tools for assessing the potential distribution of crops [14]. With the continued advancement of modeling approaches, ensemble modeling (EM), which integrates predictions from multiple machine learning algorithms, can substantially improve the accuracy and robustness of distribution predictions [15,16]. Compared with single models, ensemble approaches can effectively reduce uncertainties arising from differences in model structure, variable selection, and data quality, and provide a more comprehensive representation of crop responses to complex environmental gradients [17,18]. Similar ensemble-based SDM approaches have been extensively applied to animal species, particularly in studies of insects, where they have proven effective for predicting habitat suitability, assessing invasion risks, and evaluating species responses to climate change [19,20,21]. By integrating environmental variables with spatial crop distribution data, SDMs can simulate habitat suitability under varying environmental conditions, thereby providing a scientific basis for regional planting planning and ecological management [22]. Additionally, niche analysis provides a framework for revealing crop responses to environmental conditions and their potential migration trends [23]. By quantifying niche overlap, evaluating environmental suitability, and identifying the most dissimilar variables, it is possible to assess the reliability of projected suitable areas and detect niche shifts under future climate scenarios [24,25]. This approach not only improves our understanding of crop adaptability and potential expansion under climate change but also provides scientific support for ecological risk assessment and long-term production planning [26]. The integration of ensemble modeling with niche analysis enables a more comprehensive assessment of dynamic changes in crop suitable habitats under different climate scenarios, providing a theoretical basis for precision agricultural management and ecological conservation strategies while promoting sustainable agricultural practices in arid and semi-arid regions.

Cotton is an important economic crop in China, serving not only as a pillar industry vital to national livelihoods but also holding a strategic position in global bulk agricultural commodities and downstream textile trade. Based on cotton distribution data and multi-source environmental variables, this study integrates ensemble modeling and niche analysis to systematically assess the spatial patterns of cotton in Xinjiang and their future dynamics. The specific objectives were as follows: (1) to compare differences in suitable area and spatial distribution patterns of cotton under current conditions with and without human influences; (2) to project suitable areas under future climate scenarios and quantify their spatial changes; (3) to evaluate the applicability and similarity of future environmental conditions and identify potential limiting factors using the multivariate environmental similarity surface (MESS) and most dissimilar variable (MOD) approaches; and (4) to quantitatively assess niche overlap to reveal the ecological adaptability and potential migration capacity of cotton under climate change. The results provide high-resolution maps of cotton suitability in Xinjiang under current and future climate conditions, offering a methodological framework for spatial planning of economic crops in arid and semi-arid regions. They also provide a scientific basis for climate-adaptive management, ecological agricultural planning, and policy development, while improving our understanding of cotton’s ecological adaptation under complex environmental conditions.

## 2. Results

### 2.1. Algorithm Selection and Model Performance

**Figure 1:**
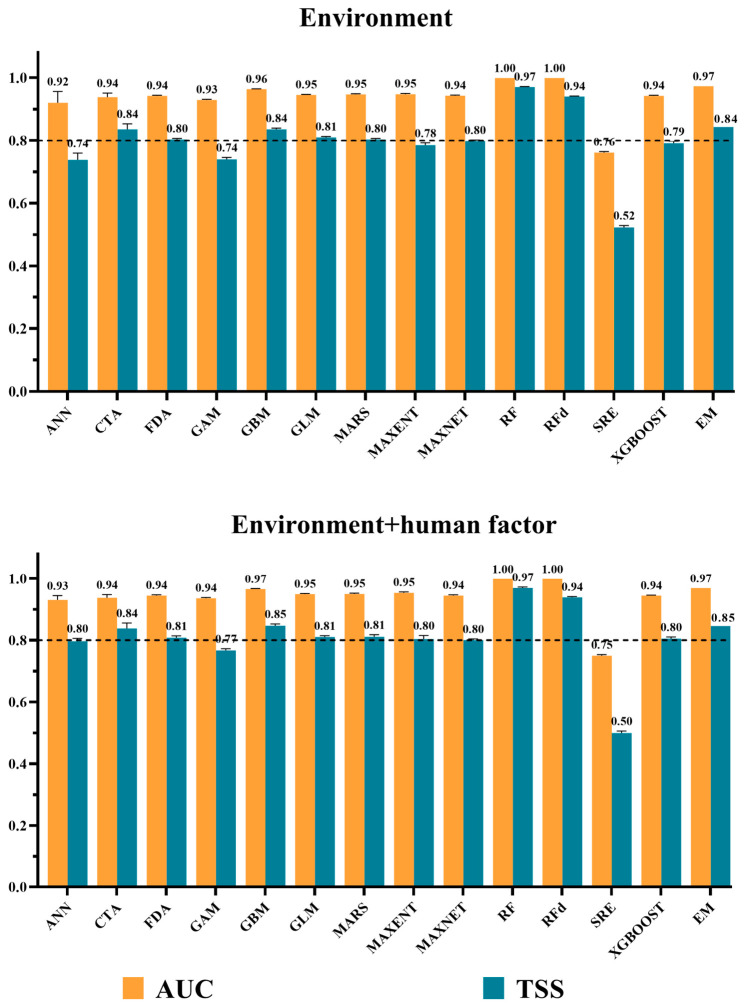
Accuracy evaluation of 13 individual and ensemble models.

In the modelling process that considered only environmental variables, CTA, FDA, GBM, GLM, MARS, MAXNET, RF and RFD were identified as meeting the requirements for ensemble modelling. The final ensemble model demonstrated high predictive accuracy, with an AUC of 0.97, a TSS of 0.84, and sensitivity and specificity of 0.97 and 0.87, respectively. In the modelling incorporating human activity factors, ANN, CTA, FDA, GBM, GLM, MARS, MAXENT, MAXNET, RF, RFD and XGBOOST were selected for inclusion in the ensemble analysis. Compared with the environment-only model, the inclusion of human factors led to a slight improvement in model performance, with the EM achieving an AUC of 0.97, a TSS of 0.85, sensitivity of 0.96, and specificity of 0.89. These results indicate that both models demonstrate strong predictive ability, while the incorporation of human activity variables further enhances model discrimination and stability (Figure 1).

### 2.2. The Importance of Driving Factors

**Figure 2:**
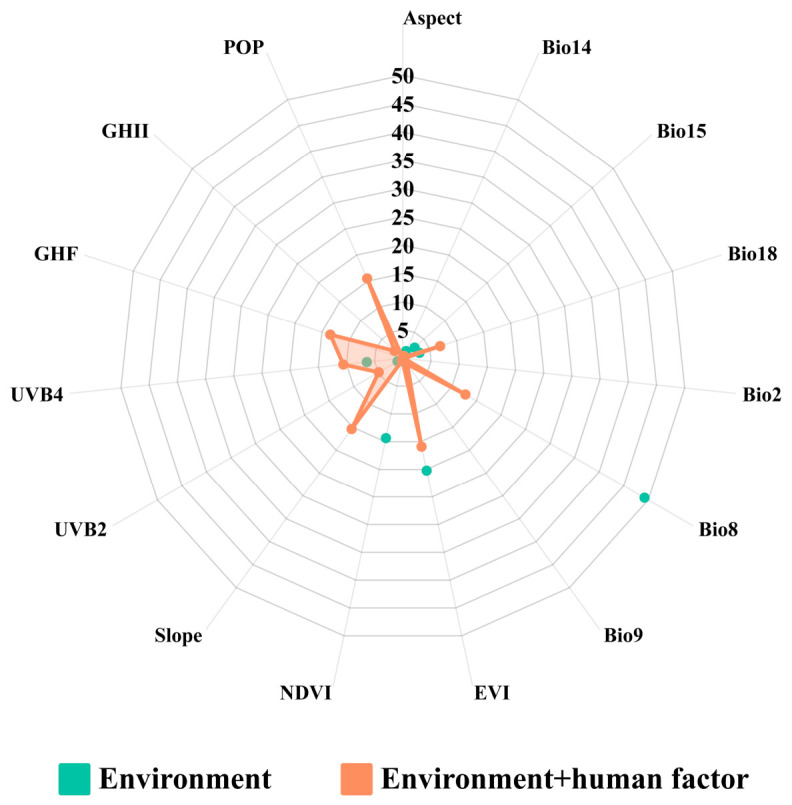
The contribution of environmental variables to the two models.

When considering environmental factors alone, the potential suitability of cotton is primarily influenced by climate and topography, with variables related to temperature and precipitation making a significant contribution (Figure 2). Among these, the mean temperature of the driest quarter makes the largest contribution (Bio9, 49.24%), followed by the normalized difference vegetation index (NDVI, 20.21%) and Slope (14.35%), whilst the contributions of precipitation seasonality (Bio15, 1.39%) and precipitation of warmest quarter (Bio18, 2.80%) were relatively minor. After incorporating human activity variables, the combined effect of environmental factors and human activities significantly altered the structure of factor contributions. The contributions of the global human footprint (GHF, 10.56%), the global human influence index (GHII, 13.46%) and population density (POP, 15.49%) increased significantly. Meanwhile, the overall importance of environmental variables declined, although Bio9 (12.74%) remained the dominant climatic factor. NDVI and EVI contributed 15.89% and 1.31%, respectively, whereas Bio2, Bio8, and Bio14 each contributed less than 7%.

In addition, response curve analysis revealed the quantitative relationship between the probability of cotton occurrence in Xinjiang and key driving factors (Figure S1). In the environment-only model, occurrence probability increased rapidly with EVI and NDVI before reaching a plateau at higher values. The influence of UVB4 and Bio18 on the probability of occurrence followed a curve trend of first rising and then falling, whilst Bio8 exhibited a continuously rising trend. After incorporating human activity variables, these response patterns were modified. Occurrence probability increased sharply with EVI and Bio8, while the effect of POP showed a slight decline at low to moderate levels, followed by a gradual increase, stabilizing between 0.7 and 0.8. Slope exhibited a weak negative relationship with occurrence probability, indicating a preference for flatter terrain. In contrast, GHF showed a unimodal response, suggesting that moderate human activity may enhance suitability through management or irrigation, whereas excessive disturbance reduces habitat suitability.

### 2.3. The Suitable Areas of Cotton Under Current and Future Climate Scenarios

**Figure 3:**
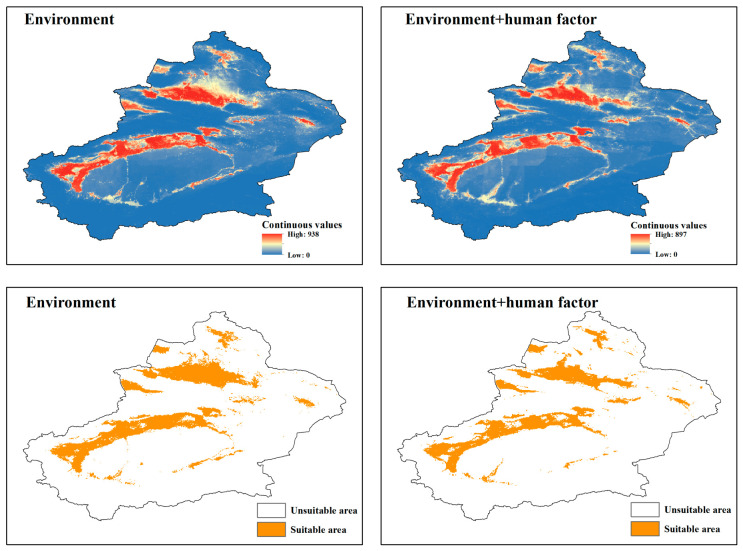
Suitable distribution areas for Xinjiang cotton under the current period.

Under environmental variables only, suitable habitats for cotton were primarily distributed across the Tarim Basin, the northern and southern slopes of the Tianshan Mountains, and the southern margin of the Junggar Basin, including major cultivation regions such as Aksu, Kashgar, Bayingolin Mongol Autonomous Prefecture, Changji, Shihezi, Wujiaqu, Karamay, Bortala Mongol Autonomous Prefecture, and Hami, with a total suitable area of 25.12 × 10^4^ km^2^ (Figure 3). After incorporating human activity factors, the suitable area decreased to 21.68 × 10^4^ km^2^, representing a 13.71% reduction compared to the environment-only model, indicating that human activities exert a constraining effect on the potential suitable habitats and negatively influence their spatial distribution (Table 1). Furthermore, with increasing greenhouse gas emissions and over time, cotton suitability is projected to expand markedly under future climate scenarios (Figure S2). The total suitable area increased across all scenarios, ranging from 28.25 to 94.10 × 10^4^ km^2^, with the most pronounced expansion occurring under the SSP5-8.5 scenario during 2061–2080 (2070s).

### 2.4. Relative Changes in Cotton Suitable Areas Under Future Climate Scenarios

**Figure 4:**
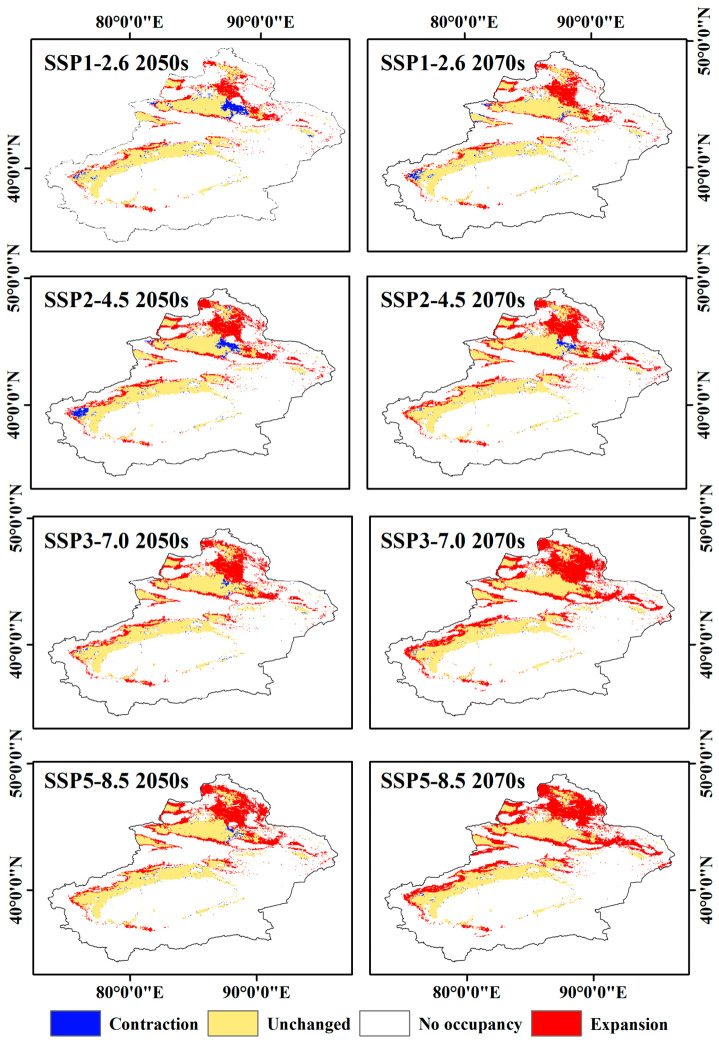
Relative areas of change for Xinjiang cotton under different future climate scenarios.

Under future climate scenarios, the suitable habitat for cotton in Xinjiang is expanding northwards (Figure 4). The area of the expanding region ranges from 9.31 to 24.01 × 10^4^ km^2^, with the largest expansion occurring under the SSP5-8.5-2070s scenario, followed by SSP3-7.0-2070s, whilst the smallest expansion is observed under the SSP1-2.6-2050s scenario. In contrast, the areas of contraction are concentrated in the western part of the Kashgar Prefecture and the Changji Hui Autonomous Prefecture. The area of the contracting regions ranges from 0.31 to 2.25 × 10^4^ km^2^, with the largest contraction occurring under the SSP2-4.5-2050s scenario, followed by SSP1-2.6-2050s, whilst the smallest contraction is observed under the SSP3-7.0-2050s scenario. It is worth noting that, under all future climate scenarios, the area of expansion exceeds that of contraction, indicating that future climate change will significantly promote the spatial expansion of the potential suitable habitat for cotton (Table 2).

### 2.5. MESS and MOD Analyses Under Future Climate Scenarios

**Figure 5:**
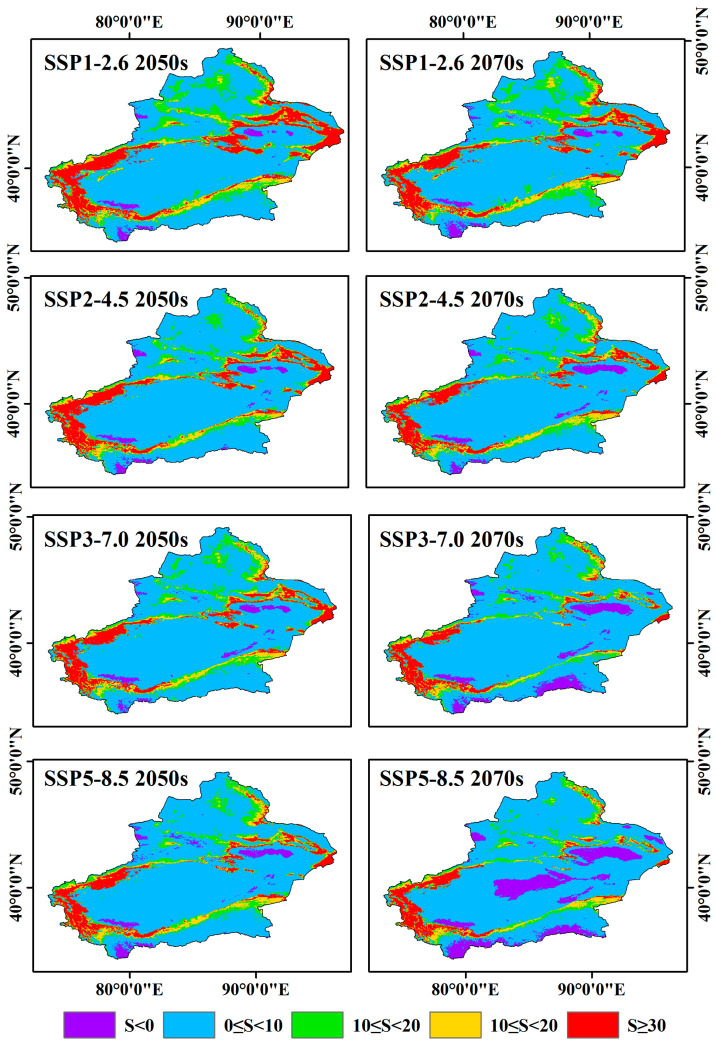
Multivariate environmental similarity surfaces (MESS) for Xinjiang cotton under different future climate scenarios.

**Figure 6:**
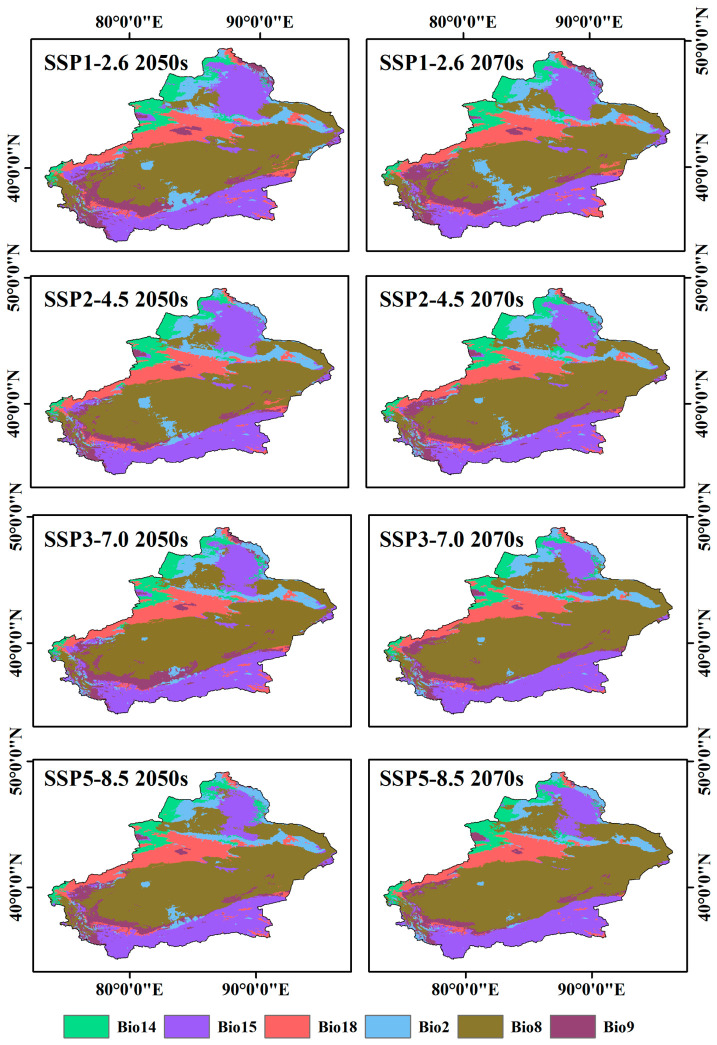
Most dissimilar variable (MOD) for Xinjiang cotton under different future climate scenarios.

The results of the MESS analysis indicate that, under future climate scenarios, areas with high similarity in bioclimatic variables are primarily distributed across western and eastern Xinjiang, including Urumqi, Hami, Aksu Prefecture, Kashgar Prefecture, and Bayingolin Mongol Autonomous Prefecture (Figure 5). These regions exhibit strong consistency between future climatic conditions and the current environmental envelope of cotton suitability, suggesting relatively stable habitat conditions under climate change scenarios. In contrast, areas with low environmental similarity are mainly concentrated in the northeastern Hami region (particularly transitional desert–oasis zones), the western part of the Bortala Mongol Autonomous Prefecture, and localized areas in the Bayingolin Mongol Autonomous Prefecture. These regions show substantial deviation from the current climatic niche, indicating potential non-analogue climate conditions in the future. Such low-similarity areas are of particular concern for cotton management, as they may experience increased climatic uncertainty and reduced reliability of suitability predictions, thereby requiring cautious evaluation before agricultural expansion or irrigation investment. Furthermore, MOD analysis indicates that temperature and precipitation are the key climatic factors influencing cotton growth and environmental adaptation (Figure 6). Among these, mean temperature of wettest quarter (Bio8) and precipitation seasonality (Bio15) are the primary driving variables responsible for environmental variations in Xinjiang, highlighting the dominant role of hydrothermal coupling in shaping cotton suitability patterns across the study region.

### 2.6. Cotton Ecological Niche Dynamics Under Future Climate Scenarios

**Figure 7:**
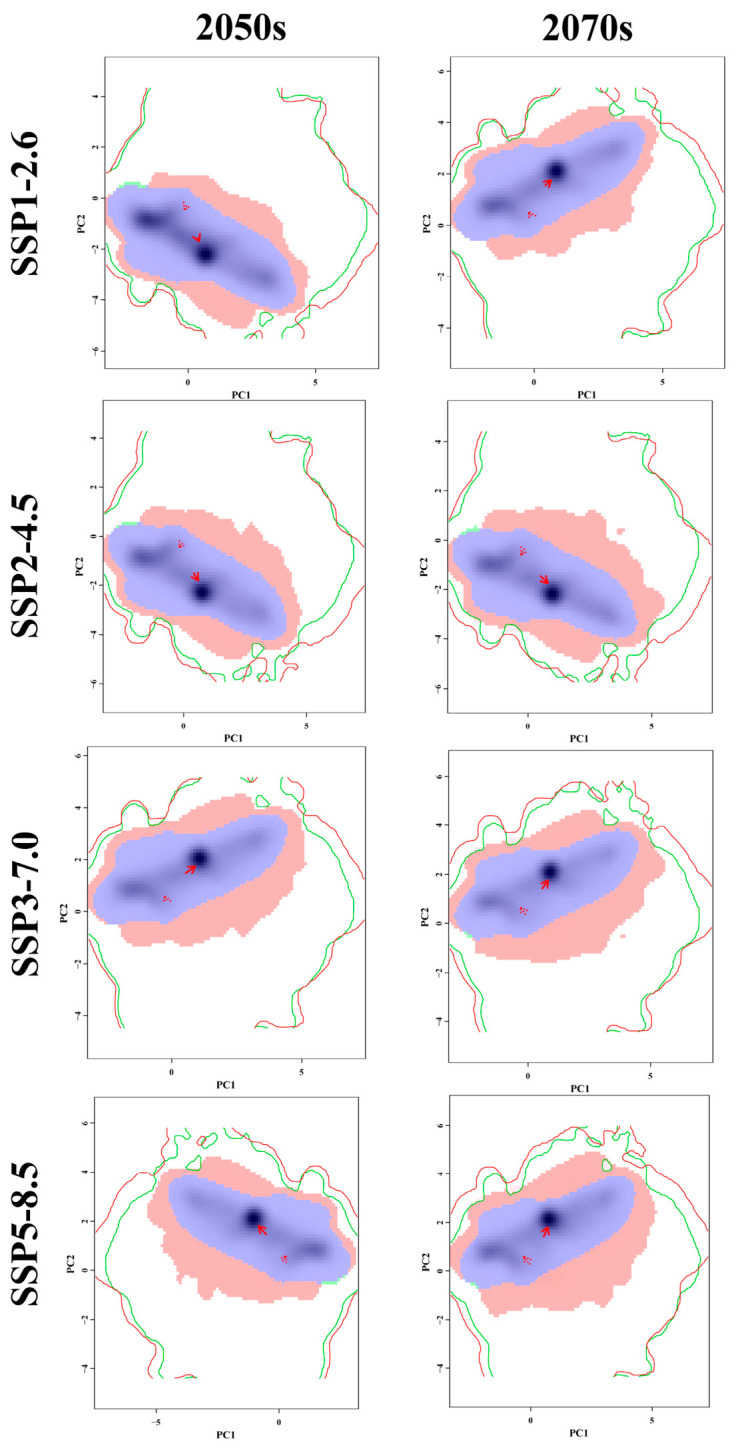
The climatic niche of Xinjiang cotton under different future climate scenarios.

Cotton is projected to maintain strong niche adaptability under future climate conditions (Figure 7). Principal component analysis (PCA) revealed that the first two components cumulatively explained 63.77% to 64.90% of the variance, indicating that the species’ niche remains relatively stable under future climate scenarios. Niche overlap indices further showed high similarity between future and current niches across different scenarios. The highest overlap occurred under SSP1-2.6 for the 2070s (Schoener’s D = 0.72, Hellinger’s I = 0.87), while the lowest was observed under SSP5-8.5 for the 2070s (D = 0.59, I = 0.78), highlighting the scenario-dependent effects of climate change on cotton’s potential suitable habitats (Table 3).

## 3. Discussion

Ensemble modeling offers clear advantages over single models, which are often constrained by algorithmic structure, underlying assumptions, and sensitivity to environmental variables, making them prone to bias or overfitting and reducing prediction accuracy and stability [15,18,27]. In contrast, an ensemble model integrates predictions from multiple algorithms, leveraging the strengths of each across diverse environmental gradients, effectively balancing model bias and variance and providing a more comprehensive representation of species responses to complex environmental conditions [19,27,28]. Moreover, by capturing prediction differences among individual models, the ensemble approach can identify areas of potential uncertainty, thereby providing more robust guidance for cultivation planning and ecological management [17,29]. For Xinjiang, a typical temperate continental arid region, environmental factors interact in complex ways, making it difficult for a single model to fully capture cotton’s responses to multidimensional environmental gradients [30,31,32]. The ensemble approach, by leveraging algorithmic complementarity, enhances the characterization of cotton’s responses to multiple drivers such as temperature, precipitation, vegetation, and human activities, thereby substantially improving model robustness and reliability [13,33]. Ensemble modeling not only increases the accuracy of crop suitability predictions but also provides a more reliable technical framework for assessing potential distribution patterns under climate change [27]. The integrated model developed in this study demonstrated high stability and discriminatory power in performance evaluation, with both models achieving an AUC value of 0.97, indicating extremely high accuracy in distinguishing between suitable and unsuitable areas for cotton cultivation. In addition, the high sensitivity and specificity indicate that the model is not only capable of effectively identifying suitable habitats but also of accurately excluding unsuitable areas, demonstrating robust predictive capabilities across different suitability gradients. However, despite these advantages, ensemble modeling also has several limitations. It typically requires higher computational resources and longer processing time due to the involvement of multiple algorithms and repeated model training. In addition, the performance of the ensemble is dependent on the quality and diversity of the included base learners. The inclusion of poorly performing or highly similar models may introduce noise and potentially reduce overall predictive efficiency. Although ensemble approaches generally reduce the risk of overfitting compared with single models, such risk may still persist under conditions of spatial autocorrelation or imbalanced sampling. Therefore, careful selection and tuning of base models remain essential to ensure model robustness and interpretability.

The suitability of Xinjiang for cotton cultivation is not determined by a single environmental factor, but rather is the combined result of climatic conditions, topographical features, vegetation cover and human activities [13,29,33,34]. Temperature and moisture conditions impose fundamental constraints on cotton growth, whilst vegetation cover and topographical conditions further influence its potential suitability by regulating the local microenvironment and resource utilization efficiency [2,35,36]. When considering environmental factors alone, temperature and precipitation-related variables constitute the primary drivers, with mean temperature of the driest quarter (Bio9) playing a dominant role, indicating that cotton is highly sensitive to temperature fluctuations under drought conditions. At the same time, high vegetation cover and gentle topography help to maintain favourable soil moisture conditions and agricultural management efficiency, thereby enhancing the suitability of the environment for cotton growth [30]. Precipitation seasonality (Bio15) and precipitation in the warmest quarter (Bio18) exert an indirect regulatory effect on soil moisture and the crop growth environment by influencing the spatio-temporal distribution patterns of water [5,37]. Response curves further reveal the non-linear characteristics of these key factors’ influence on cotton suitability. The probability of cotton occurrence rises rapidly and then stabilises with increasing EVI and NDVI, indicating that areas with high vegetation cover typically possess more favourable light conditions and soil environments [2]. Meanwhile, mean temperature of wettest quarter (Bio8) exhibits a consistent positive relationship, further emphasising the promoting effect of warm and humid conditions on cotton growth [38]. Following the incorporation of human activity factors, the driving mechanisms underwent a marked adjustment, reflecting a significant interactive effect between human management and the natural environment [39]. The contributions of population density (POP), global human activity footprint (GHF) and global human impact index (GHII) increased significantly, indicating that moderate human intervention (such as irrigation, fertilization and agricultural management measures) can, to a certain extent, alleviate environmental constraints and enhance the suitability of cotton in marginal areas. However, when the intensity of human activity exceeds a certain threshold, it may exert a negative impact on cotton growth through mechanisms such as increased water consumption and soil structure degradation [30,39]. Meanwhile, although the relative contributions of environmental factors have declined, Bio9 continues to play a key driving role, whilst the sustained positive growth of NDVI and EVI further demonstrates the central regulatory function of vegetation conditions in the formation of suitability. Furthermore, the negative impact of slope on the probability of cotton occurrence reflects the significant advantages of gently sloping areas in terms of soil and water conservation and mechanized production, which are more conducive to stable agricultural production [27]. In summary, cotton suitability in Xinjiang is jointly regulated by multiple environmental gradients and human activities, with the relative influence of each factor varying across different modeling frameworks. This finding underscores the need, when planning cotton cultivation in arid and semi-arid regions, to take a holistic view of climatic conditions, topographical features and human interventions, and to achieve the rational utilization of suitable areas and the harmonious development of the ecological environment through the optimization of resource allocation and management measures.

The potential suitability of cotton cultivation in Xinjiang exhibits distinct geographical patterns, being concentrated primarily in typical growing regions such as the Tarim Basin north and south of the Tianshan Mountains and the southern edge of the Junggar Basin [40,41]. Suitability in these areas is determined by the combined effects of temperature, precipitation, vegetation cover and topographical conditions, resulting in a spatial distribution pattern shaped by multi-dimensional environmental constraints [37,42,43,44]. Incorporating human activity factors led to a reduction in the area of suitable habitats, indicating that population distribution, land development, and infrastructure construction exert a constraining effect on the potential distribution of cotton. This suggests that considering only natural environmental conditions may overestimate the potential suitable area, highlighting the necessity of integrating human interventions for a comprehensive assessment in cotton production planning. Under future climate scenarios, the potential suitable area for cotton generally shows a trend of expansion northwards. This spatial pattern reflects the promoting effect of improved temperature and moisture conditions on cotton growth against a backdrop of climate warming, whilst also indicating that mid-to-high latitude regions may become important areas for future potential suitable habitats [45,46,47]. In contrast, the areas of contraction are mainly concentrated in the western part of the Kashgar Prefecture and the Changji Hui Autonomous Prefecture, suggesting that local environmental pressures may still restrict cotton growth. It is worth noting that, under all future emission scenarios, the expanding areas consistently exceed the contracting areas, suggesting that future climate change will, on the whole, increase the spatial coverage of Xinjiang’s potential suitable cotton-growing areas. Therefore, future agricultural planning should prioritize resource allocation and ecological management in northern Xinjiang and other potential expansion areas. Cotton planting should be strategically arranged by considering water availability, soil conditions, and current land use, to fully capitalize on opportunities presented by climate change and enhance production stability and sustainability [48,49]. At the same time, the negative impacts of human activities on suitable habitats highlight the importance of careful planning and management. This indicates that cotton growers and policymakers can optimize land use to mitigate excessive disturbances, ensuring that the potential benefits of habitat expansion are effectively translated into actual production gains. It should be noted that the effects of human activities and climate change operate through different mechanisms. Human activity primarily imposes a spatial constraint on current suitable habitats through land-use intensity, population density, and infrastructure development, thereby reducing realized suitability under present conditions. In contrast, climate change-driven shifts reflect changes in climatic suitability and potential ecological niches under future scenarios. Therefore, human activities do not directly alter the overall trend of climate-induced expansion, but they can significantly reshape its spatial realization by restricting expansion in highly developed or intensively used areas, while relatively less disturbed regions remain more responsive to climate-driven suitability gains.

MESS and MOD analyses have revealed the underlying environmental conditions and limiting factors for the potential future distribution of cotton in Xinjiang. Although future climate change may exert some influence on the cotton growing environment, most areas in western and eastern Xinjiang will retain a high degree of environmental similarity, indicating that these regions will continue to possess strong potential suitability in the future. In particular, the major cotton-growing regions north and south of the Tianshan Mountains exhibit climatic conditions that are highly consistent with current suitable habitats, providing a reliable environmental foundation for the future expansion of cotton cultivation. In contrast, Turpan, the western part of the Bortala Mongol Autonomous Prefecture, and certain localized areas of Kunyu exhibit lower environmental similarity, suggesting that climate change may constrain cotton growth and distribution in these regions. MOD analysis further indicates that temperature and precipitation are the core climatic factors influencing cotton’s ecological adaptation. Mean temperature of the wettest quarter (Bio8) and precipitation seasonality (Bio15) contribute most significantly to regional environmental variations. This indicates that cotton distribution is influenced not only by annual mean temperature and total precipitation but also, to a greater extent, by the seasonal characteristics of temperature and precipitation during the wet season [50,51]. In addition, ecological niche stability analysis further revealed the adaptive potential of cotton in Xinjiang under future climate change. Future climate scenarios may lead to localized changes in suitable habitats, but cotton is expected to retain a relatively stable ecological niche. PCA results indicate that the first two principal components consistently account for approximately 64% of the total variance, highlighting the persistent influence of major environmental gradients on cotton’s niche. Niche overlap indices further demonstrate that future cotton niches remain largely consistent with the current niche, suggesting that cotton in Xinjiang retains strong climatic adaptability and is likely to sustain habitat suitability under projected climate conditions. These findings suggest that in future potential suitable areas and marginal expansion zones, appropriate irrigation, temperature control and cultivation management measures can mitigate climatic constraints and enhance the stability of cotton growth. Consequently, high-similarity regions in northern and western Xinjiang can be prioritized for expansion, whilst targeted management should be implemented in low-similarity regions to achieve climate-adaptive cultivation and optimize production stability.

## 4. Materials and Methods

### 4.1. Data on the Distribution of Cotton

**Figure 8:**
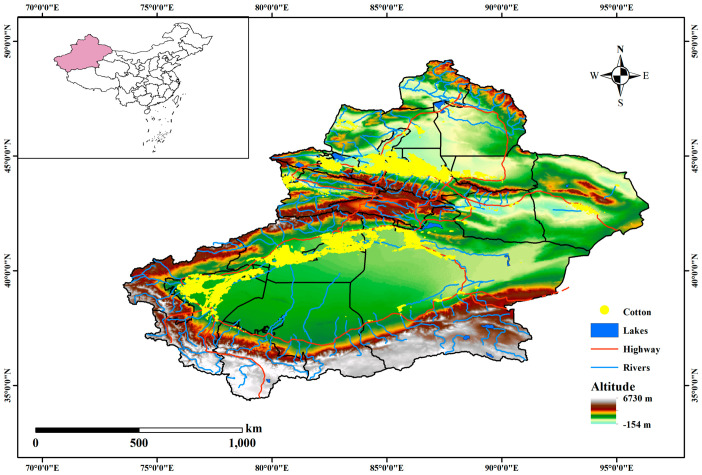
Distribution of cotton cultivation in Xinjiang.

Cotton distribution data were obtained from the 10 m resolution cotton mapping dataset for Xinjiang published by Kang et al. (2023) [8] on the Zenodo platform (https://zenodo.org/records/7856467, accessed on 30 January 2026). The 2021 dataset was selected as the baseline, and its high spatial resolution allows for a detailed representation of cotton planting patterns in the study area [8]. During preprocessing, cotton planting information was extracted from each data tile, followed by mosaicking and integration of raster layers under a unified coordinate reference system to generate a continuous cotton distribution dataset covering the entire Xinjiang region (Figure 8).

To reduce overfitting caused by spatial autocorrelation, spatial thinning was performed using the spThin package (v 0.2.0) in R (v 4.4.1). A thinning distance of 2.5 arc-minutes (approximately 4.6 km) was applied, consistent with the spatial resolution of the environmental variables (2.5 arc-minutes), ensuring that only one occurrence point was retained within each environmental grid cell. This approach reduced redundancy in environmental information and minimized spatial autocorrelation effects. After thinning, the remaining occurrence records were still sufficient for robust model training and validation. The processed dataset improved data quality and reduced potential biases associated with spatial clustering, thereby enhancing the accuracy and robustness of model predictions [52].

### 4.2. Sources and Filtering of Environment Variables

The bioclimatic data used in this study were obtained from the WorldClim database (v 2.1; https://www.worldclim.org/, accessed on 20 January 2025), including 19 current bioclimatic variables at a spatial resolution of 2.5 arc-minutes. These variables encompass the annual average characteristics and seasonal variations of temperature and precipitation, and are effective in characterising species’ responses to climatic conditions [53,54]. Future climate data were selected from the BCC-CSM2-MR model within the CMIP6 framework, covering two periods: 2041–2060 (2050s) and 2061–2080 (2070s). Four Shared Socioeconomic Pathways (SSP) scenarios were employed, including the low-emission scenario (SSP1-2.6), medium-emission scenarios (SSP2-4.5 and SSP3-7.0) and high-emission scenarios (SSP5-8.5), to reflect climate change trends under different socio-economic development pathways. Vegetation indices and elevation data were obtained from the Resource and Environmental Science Data Platform (https://www.resdc.cn, accessed on 20 January 2026). Concurrently, slope and aspect were calculated based on elevation using ArcGIS Map (v10.8.1). Solar radiation data were sourced from the Helmholtz Centre for Environmental Research (https://www.ufz.de, accessed on 15 January 2026). Furthermore, variables related to human activities were downloaded from the Socioeconomic Data and Applications Centre (https://www.unccd.int/resources/knowledge-sharing-system/socio-economic-data-applications-center-sedac, accessed on 15 January 2026). Finally, all environmental variables were uniformly processed using resampling and spatial clipping to ensure consistency across all raster data in terms of spatial resolution, coordinate systems and spatial extent.

Environmental variables often exhibit strong correlations, which can induce spatial autocorrelation and multicollinearity, thereby reducing the stability of model parameter estimates and the reliability of predictions [55]. To reduce variable redundancy, this study utilized the usdm package (v2.1.7) to perform Pearson correlation analyses (r < 0.8) on 33 environmental variables in order to identify highly correlated variables. Building on this, a stepwise screening process using the variance inflation factor (VIF) was conducted, sequentially excluding variables with VIF values greater than 10 to mitigate the impact of multicollinearity (Figure S1). This screening process effectively reduced the degree of linear dependence among environmental variables, ensuring the relative independence of the variable set and thereby enhancing the model’s robustness and predictive accuracy. Ultimately, 15 key environmental factors were selected for subsequent modelling and analysis (Table 4).

### 4.3. Construction and Integration of SDM

This study utilizes the Biomod2 package (v 4.2-6) to model the distribution of cotton in Xinjiang, employing an ensemble modelling approach to combine the predictions of multiple individual algorithms in order to enhance the overall accuracy and robustness of the forecasts [22,28]. The selected algorithms included artificial neural networks (ANN), classification tree analysis (CTA), flexible discriminant analysis (FDA), generalized additive models (GAM), generalized boosting models (GBM), generalized linear models (GLM), multivariate adaptive regression splines (MARS), maximum entropy via MAXNET (MAXNET), random forest (RF), surface range envelope (SRE), and extreme gradient boosting (XGBOOST). This multi-algorithm approach allows the model to capture diverse relationships between cotton distribution and environmental predictors while leveraging the complementary strengths of different modeling techniques.

Model predictive performance was evaluated using the area under the receiver operating characteristic curve (AUC) and the true skill statistic (TSS). AUC values measure the model’s ability to discriminate between the presence and absence of cotton, ranging from 0 to 1, with higher values indicating stronger discriminative power [56]. TSS values combine sensitivity and specificity, ranging from −1 to 1, with values closer to 1 indicating higher predictive reliability [57]. By employing both AUC and TSS values, the model’s accuracy and robustness can be comprehensively assessed from multiple perspectives.

To ensure the robustness of the ensemble model, only individual models with AUC > 0.9 and TSS > 0.8 were retained for integration. During modeling, 80% of occurrence records were used as the training set and the remaining 20% as the test set, while 10,000 pseudo-absence points were randomly generated to balance the sample distribution. Each algorithm was run 10 times with 10-fold cross-validation to reduce model uncertainty and variance. In addition, Biomod2’s automated tuning function (tuned) was applied to optimize model parameters and further improve predictive performance. Finally, eligible single-model outputs were integrated using the Weighted Mean (Wmean) method to generate a high-accuracy, robust prediction map of potential cotton distribution, providing a reliable basis for subsequent habitat suitability analyses [55].

### 4.4. Binary Classification of Cotton Suitability

This study developed 2 types of models (Model 1: Environmental variables only; Model 2: Environmental + human activity variables) and constructed 3 analytical schemes (Scheme 1: Current environmental variables; Scheme 2: Current environmental variables combined with human activity; Scheme 3: Future environmental variables) to systematically evaluate the effects of environmental factors and human activities on cotton suitability in Xinjiang. Scheme 1 and Scheme 2, both based on current climatic conditions, were used to quantify the regulatory effects of human activities on habitat suitability, whereas Scheme 1 and Scheme 3 were compared to assess the potential impacts of future climate change on cotton distribution [58].

The optimized ensemble model was used to predict the potential distribution of cotton. Model outputs were generated as continuous raster layers (TIFF), in which each grid cell represents habitat suitability probability (*p*) ranging from 0 to 1000, with higher values indicating more favorable environmental conditions. For quantitative analysis and visualization, the optimal threshold was determined based on the TSS value to convert continuous suitability maps into binary classifications, thereby delineating suitable and unsuitable habitats and providing a clear reference for ecological management and cultivation planning. In addition, to assess dynamic changes in suitable habitats under future climate scenarios, the BIOMOD_RangeSize function was employed to quantify spatial shifts in suitability. Changes were categorized into 4 types: Expansion (increase in distribution area), contraction (decrease in distribution area), unchanged (no change), and no occupancy (not occupied under future scenarios). This approach enables a clear characterization of spatial dynamics in cotton distribution across different climate scenarios and provides a scientific basis for optimizing future planting strategies and ecological management [18,55].

### 4.5. Assessment of the Applicability and Similarity of Environmental Variables

The multivariate environmental similarity surface (MESS) and most dissimilar variable (MOD) approaches were employed to evaluate the applicability of environmental variables under future climate scenarios and to identify potential environmental anomalies within predicted cotton distribution areas [59]. In the MESS analysis, environmental conditions in the current suitable habitat were used as a reference to calculate the similarity index (S) between future and baseline conditions. Positive values (S > 0) indicate that future environmental conditions are within the range of the reference environment, whereas negative values (S < 0) indicate novel conditions that deviate from the current range and may constrain the expansion of cotton distribution. MESS analysis can effectively identify areas of environmental novelty within potential suitable habitats, thereby enabling an assessment of the reliability of the model’s predictions. The MOD analysis was further used to quantify which environmental variables contribute most to the observed dissimilarities under future scenarios, thereby identifying key limiting factors affecting cotton suitability. The output results for both MESS and MOD were generated using the density.tools.Novel function implemented in maxent.jar [60].

### 4.6. Niche Overlap in Environmental Variables

To quantitatively assess the degree of niche overlap for cotton under current and future climate scenarios, this study utilized the ecospat package (v 4.0.0) to calculate principal component analysis (PCA-env), Schoener’s D index and Hellinger’s I index [23,24,25]. The PCA-env method reduces the dimensionality of multiple environmental variables to two principal components, revealing the distribution patterns of cotton in a multidimensional climatic space and its potential shifting trends in response to climate change. Schoener’s D index and Hellinger’s I index are used to quantify the degree of niche overlap, with values ranging from 0 to 1, where 0 indicates no niche overlap and 1 indicates complete overlap. By combining these three indicators, this study is able to systematically assess the impact of climate change on the stability of cotton’s ecological niche, providing a scientific basis for understanding cotton’s adaptability and distribution expansion.

## 5. Conclusions

This study systematically evaluated the spatial patterns and potential shifts of cotton suitability in Xinjiang under climate change and human activity pressures using ensemble modeling combined with ecological niche analysis. Our results indicate that climate change is likely to expand suitable habitats for cotton, but the distribution patterns are strongly modulated by temperature, water availability, and anthropogenic factors, exhibiting clear regional variation and scenario dependence. Under future climate scenarios, cotton continues to occupy a relatively stable ecological niche, with niche overlap indices remaining at moderately high levels (Schoener’s D = 0.59–0.72; Hellinger’s I = 0.78–0.87), suggesting high niche conservatism with only moderate spatial redistribution under climate change. These findings reveal the mechanisms by which crops in arid and semi-arid regions respond to multidimensional environmental gradients and highlight the robustness of ensemble modeling in integrating multiple algorithms and reducing prediction uncertainty. From an applied perspective, future agricultural planning in Xinjiang should consider climate trends and potential suitability patterns to optimize cotton planting layouts, concentrate production in highly suitable areas, and adjust planting intensity in marginal zones. At the same time, strengthening water resource management and implementing adaptation measures will further enhance the resilience of the cotton production system to climate change. Overall, this study provides a high-resolution assessment of cotton suitability under current and future climates, offering both methodological guidance and theoretical insight for the sustainable management of cotton and other industrial crops in arid and semi-arid regions.

## Figures and Tables

**Table 1:** Suitable area for Xinjiang cotton under current and different future climate scenarios.

Shared Socioeconomic Pathways	Predicted Area (×10^4^ km^2^)		Comparison with Current Period Distribution (%)	
	Unsuitable Area	Suitable Area	Unsuitable Area	Suitable Area
Current-Environment	150.00	25.12	-	-
Current-Environment + human factor	153.45	21.68	2.3	−13.71
Future-SSP1-2.6 2041–2060	142.91	32.22	−4.73	28.25
Future-SSP1-2.6 2061–2080	139.63	35.50	−6.92	41.32
Future-SSP2-4.5 2041–2060	137.38	37.74	−8.42	50.26
Future-SSP2-4.5 2061–2080	135.23	39.90	−9.85	58.83
Future-SSP3-7.0 2041–2060	135.14	39.98	−9.91	59.16
Future-SSP3-7.0 2061–2080	127.20	47.92	−15.20	90.76
Future-SSP5-8.5 2041–2060	132.75	42.38	−11.50	68.69
Future-SSP5-8.5 2061–2080	126.36	48.76	−15.76	94.10

**Table 2:** Relative spatial changes for Xinjiang cotton under different future climate scenarios.

Shared Socioeconomic Pathways	Predicted Area (×10^4^ km^2^)			
	Contraction	Unchanged	No Occupancy	Expansion
Future-SSP1-2.6 2041–2060	2.22	22.90	140.69	9.31
Future-SSP1-2.6 2061–2080	1.35	23.77	138.27	11.73
Future-SSP2-4.5 2041–2060	2.25	22.87	135.13	14.88
Future-SSP2-4.5 2061–2080	1.05	24.07	134.17	15.83
Future-SSP3-7.0 2041–2060	0.81	24.31	134.33	15.67
Future-SSP3-7.0 2061–2080	0.31	24.81	126.89	23.11
Future-SSP5-8.5 2041–2060	0.68	24.44	132.07	17.94
Future-SSP5-8.5 2061–2080	0.37	24.75	125.99	24.01

**Table 3:** Principal component contributions and niche overlap indices of Xinjiang cotton under different future climate scenarios.

Shared Socioeconomic Pathways	PC1 (%)	PC2 (%)	Cumulative PC (%)	Schoener’s Index (D)	Hellinger’s Index (I)
Future-SSP1-2.6 2041–2060	34.74	30.16	64.90	0.71	0.85
Future-SSP1-2.6 2061–2080	34.68	30.09	64.77	0.72	0.87
Future-SSP2-4.5 2041–2060	34.41	30.32	64.73	0.69	0.84
Future-SSP2-4.5 2061–2080	34.34	29.96	64.30	0.66	0.82
Future-SSP3-7.0 2041–2060	34.42	30.01	64.43	0.68	0.84
Future-SSP3-7.0 2061–2080	33.88	29.96	63.84	0.63	0.81
Future-SSP5-8.5 2041–2060	34.18	30.26	64.44	0.67	0.84
Future-SSP5-8.5 2061–2080	33.70	30.07	63.77	0.59	0.78

**Table 4:** Filtering of environment variables.

Types	Abbreviation	Environmental Variables	Operation (|*r*| < 0.8)
Bioclimate	Bio1	Annual mean temperature (°C)	Eliminate
	Bio2	Mean diurnal range (°C)	Retain
	Bio3	Isothermality	Eliminate
	Bio4	Temperature seasonality	Eliminate
	Bio5	Maximum temp of warmest month (°C)	Eliminate
	Bio6	Minimum temp of coldest month (°C)	Eliminate
	Bio7	Temperature annual range (°C)	Eliminate
	Bio8	Mean temp of wettest quarter (°C)	Retain
	Bio9	Mean temp of driest quarter (°C)	Retain
	Bio10	Mean temp of warmest quarter (°C)	Eliminate
	Bio11	Mean temp of coldest quarter (°C)	Eliminate
	Bio12	Annual precipitation (mm)	Eliminate
	Bio13	Precipitation of wettest month (mm)	Eliminate
	Bio14	Precipitation of driest month (mm)	Retain
	Bio15	Precipitation seasonality (mm)	Retain
	Bio16	Precipitation of wettest quarter (mm)	Eliminate
	Bio17	Precipitation of driest quarter (mm)	Eliminate
	Bio18	Precipitation of warmest quarter (mm)	Retain
	Bio19	Precipitation of coldest quarter (mm)	Eliminate
Topography	Altitude	Elevation (m)	Eliminate
	Aspect	Aspect	Retain
	Slope	Slope	Retain
Radiation	UVB1	Annual mean UV-B	Eliminate
	UVB2	UV-B seasonality	Retain
	UVB3	Mean UV-B of highest month	Eliminate
	UVB4	Mean UV-B of lowest month	Retain
	UVB5	Sum of UV-B radiation of highest quarter	Eliminate
	UVB6	Sum of UV-B radiation of lowest quarter	Eliminate
Vegetation	NDVI	Normalized difference vegetation index	Retain
	EVI	Enhanced vegetation index	Retain
Human activities	GHF	Global human footprint	Retain
	GHII	Global human influence index	Retain
	POP	Population density	Retain

## Data Availability

Data in this study are available from the corresponding author.

## Supplementary Materials

The following supporting information can be downloaded at https://www.mdpi.com/article/10.3390/plants15111622/s1. Figure S1: Variance inflation factors (A) and correlation coefficients (B) of environmental variables; Figure S2: Response curves for environmental factors and human activities; Figure S3: Habitat suitability of cotton in Xinjiang under different future climate scenarios.
