# Supplementary material for: Human Activities Have Reduced the Potential Distribution of Cotton in Xinjiang, but Climate Change Is Expected to Expand Its Future Suitable Area
Source: Plants (Basel). 2026 May 25;15(11):1622. doi: 10.3390/plants15111622 (PMC13258848; doi:10.3390/plants15111622)
Supplement: Supplementary file 1 [file plants-15-01622-s001.zip › plants-4316396-supplementary.pdf]

Climate change and human activities jointly shape cotton suitability in Xinjiang: Insights from ensemble modeling and niche analysis

Supplementary Materials

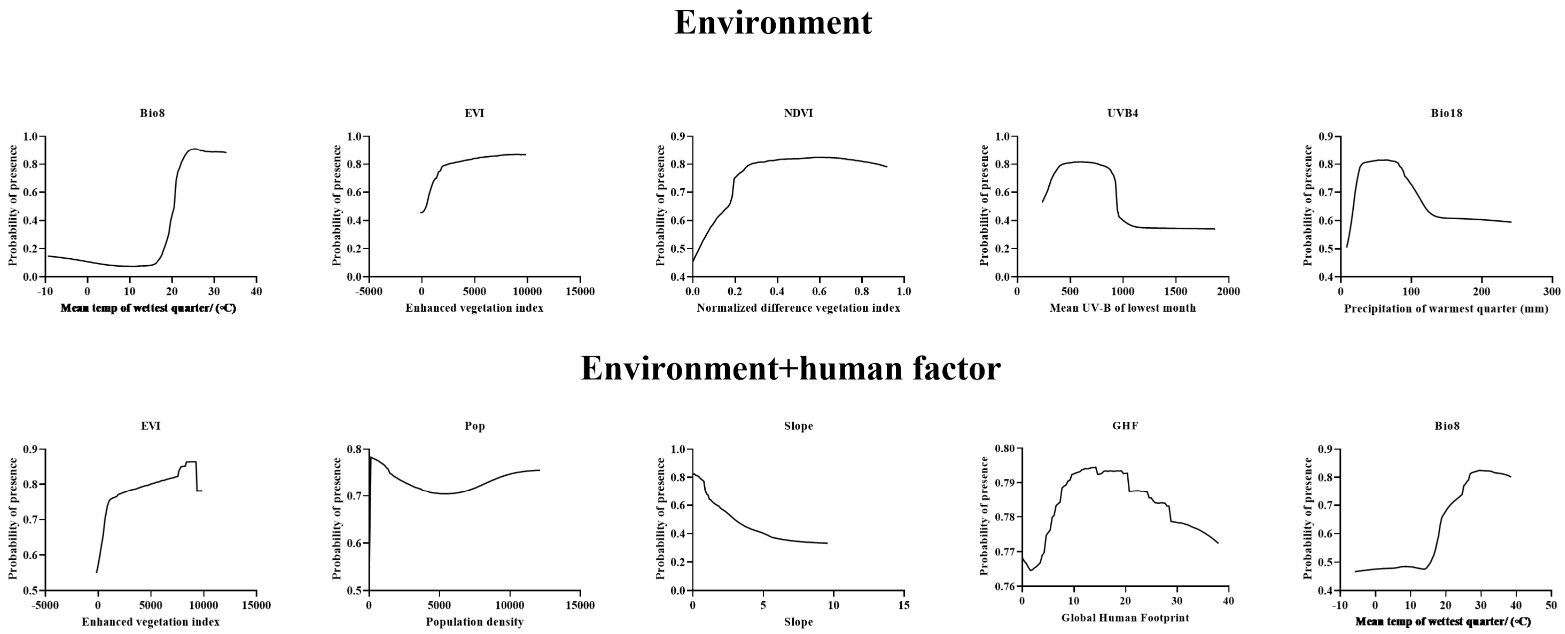

Figure S1. Response curves for environmental factors and human activities.

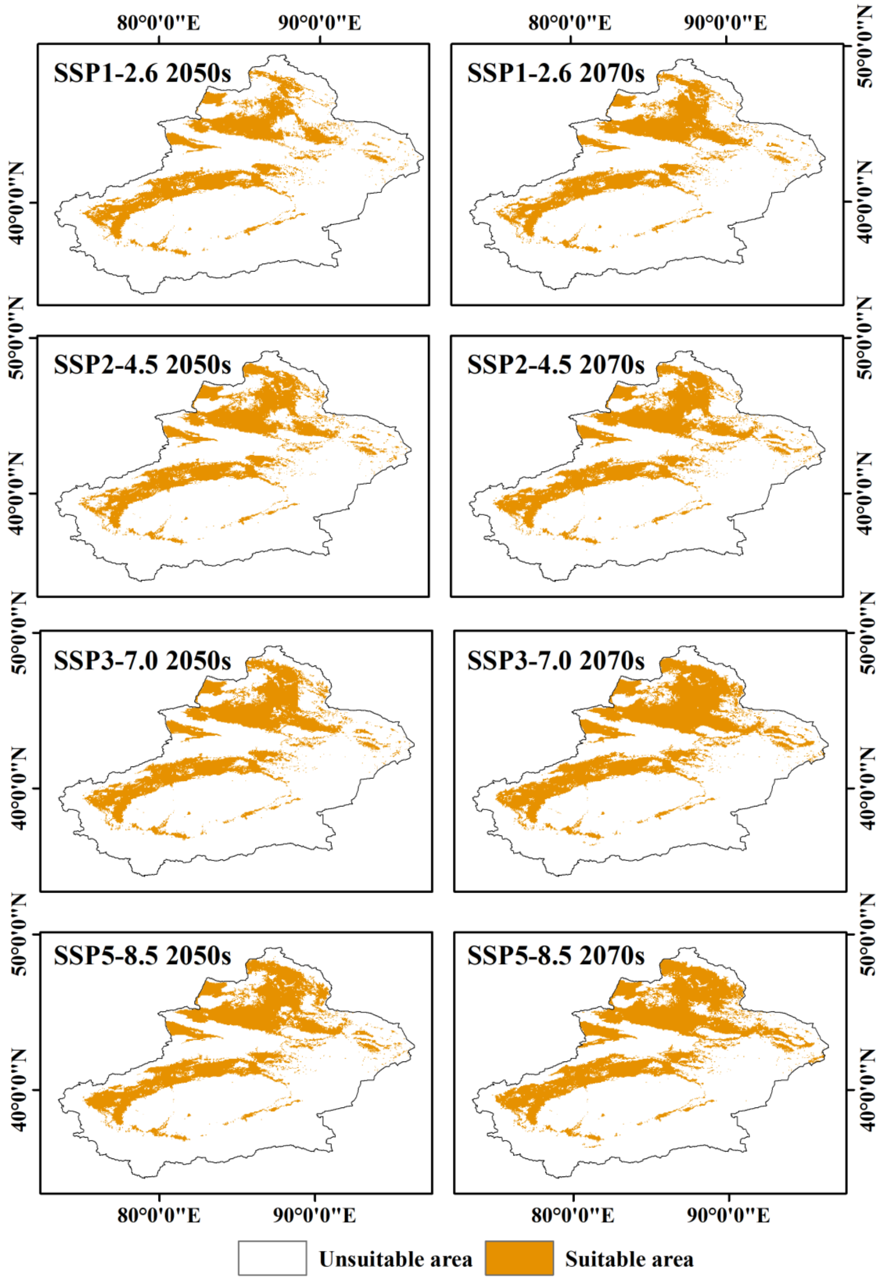

Figure S2. Habitat suitability of cotton in Xinjiang under different future climate scenarios.

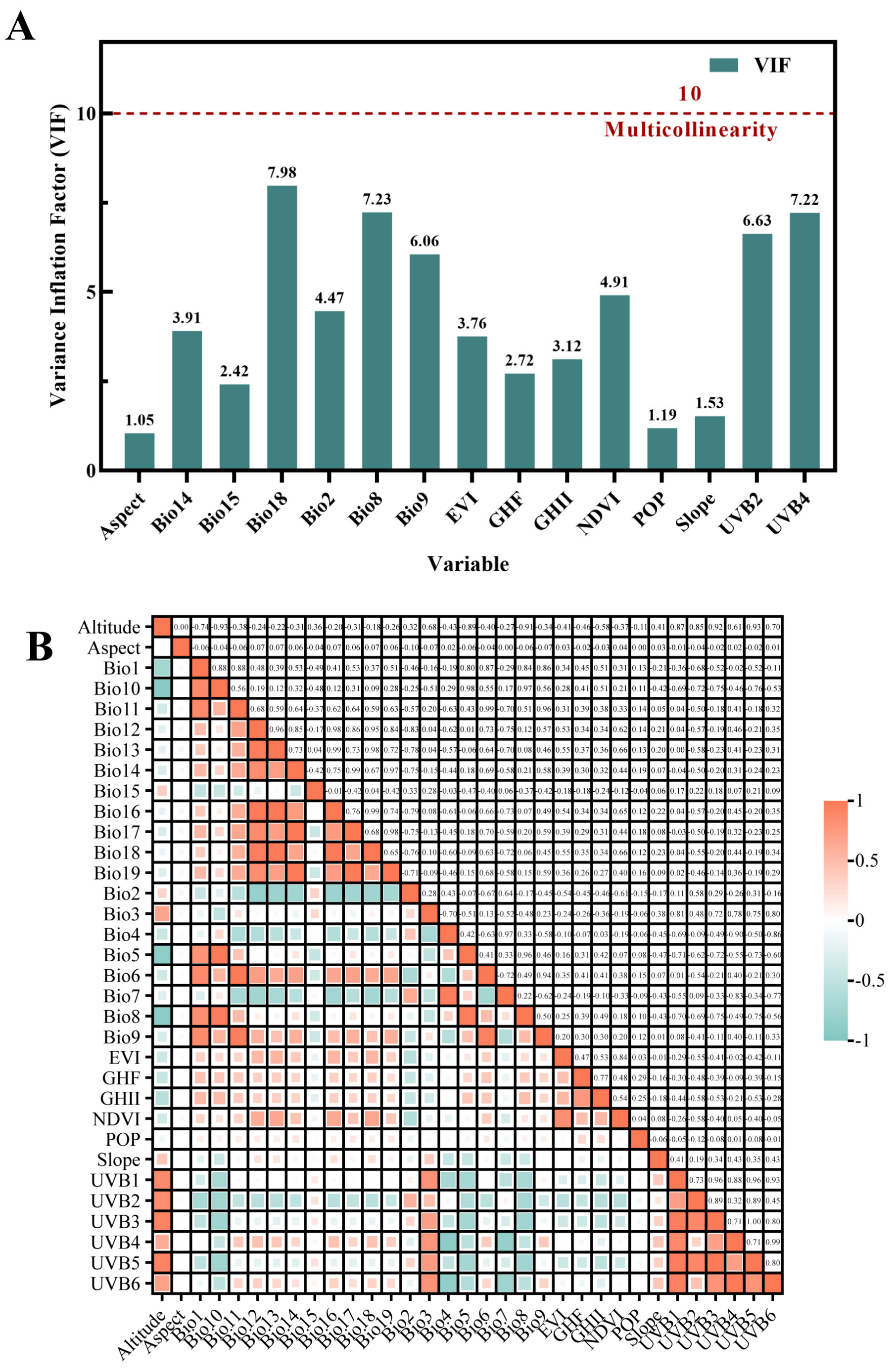

Figure S3. Variance inflation factors (A) and correlation coefficients (B) of environmental variables.
